# Characterization of the beta amyloid precursor protein-like gene in the central nervous system of the crab *Chasmagnathus*. Expression during memory consolidation

**DOI:** 10.1186/1471-2202-11-109

**Published:** 2010-09-01

**Authors:** Maria Sol Fustiñana, Pablo Ariel, Noel Federman, Ramiro Freudenthal, Arturo Romano

**Affiliations:** 1 Laboratorio de Neurobiología de la Memoria, Departamento de Fisiología, Biología Molecular y Celular, Facultad de Ciencias Exactas y Naturales, Universidad de Buenos Aires. IFIByNE, CONICET. Ciudad Universitaria, Pab. II, 2do piso (1428EHA). Buenos Aires, Argentina

## Abstract

**Background:**

Human β-amyloid, the main component in the neuritic plaques found in patients with Alzheimer's disease, is generated by cleavage of the β-amyloid precursor protein. Beyond the role in pathology, members of this protein family are synaptic proteins and have been associated with synaptogenesis, neuronal plasticity and memory, both in vertebrates and in invertebrates. Consolidation is necessary to convert a short-term labile memory to a long-term and stable form. During consolidation, gene expression and *de novo *protein synthesis are regulated in order to produce key proteins for the maintenance of plastic changes produced during the acquisition of new information.

**Results:**

Here we partially cloned and sequenced the beta-amyloid precursor protein like gene homologue in the crab *Chasmagnathus *(cappl), showing a 37% of identity with the fruit fly *Drosophila melanogaster *homologue and 23% with *Homo sapiens *but with much higher degree of sequence similarity in certain regions. We observed a wide distribution of cappl mRNA in the nervous system as well as in muscle and gills. The protein localized in all tissues analyzed with the exception of muscle. Immunofluorescence revealed localization of cAPPL in associative and sensory brain areas. We studied gene and protein expression during long-term memory consolidation using a well characterized memory model: the context-signal associative memory in this crab species. mRNA levels varied at different time points during long-term memory consolidation and correlated with cAPPL protein levels

**Conclusions:**

cAPPL mRNA and protein is widely distributed in the central nervous system of the crab and the time course of expression suggests a role of cAPPL during long-term memory formation.

## Background

Human β-amyloid precursor protein (APP) has been the subject of intense study since it was determined that it is the precursor of the β-amyloid, the main component of neuritic plaques found in patients with Alzheimer's disease (AD). Important efforts have been made to investigate its neurotoxic effects in relation with its aggregation properties and its overexpression [1,2].

Beyond the pathogenic role of βA in humans, in the last 10 years a notable convergence of results point to the role of APP holoprotein and its fragments in synaptic function. APP is able to modulate a wide variety of neuronal responses such as neuritic growth, synaptogenesis, synaptic plasticity and neuronal protection to excitotoxic damage [3-12]. Based on the general structure of the protein and the conservation of different domains it has been suggested that APP could function as a receptor [13], a growth factor [14,15] and a cell-cell or cell-substrate adhesion molecule [16,17]. Administration of different peptides derived from the cleavage of APP modulates memory in a positive as well as in a negative way, both in vertebrates and invertebrates [18-23]. In addition, KO animals show memory deficits [24]. Nevertheless, the precise physiological function of APP and its fragments remains to be clarified.

APP protein belongs to a transmembrane protein family (integrated membrane proteins type I). Homologous genes with conserved domains have been found in invertebrates: appl in *Drosophila melanogaster *[25,26]), appl in *Manduca sexta *[27], app in squid ([28]) and apl-1 in *Caenorhabditis elegans *[29]. The homologous genes in *Drosophila *and *Caenorhabditis *are unique in their respective genomes, at variance with vertebrates in which more than one member of this family is present. In mammals, there is one APP gene and two homologous genes (APP-like) called APLP-1 y APLP-2 [16]. Mammals' APP and APPL are localized in different tissues. However, *Drosophila *APPL expression is fundamentally in the central nervous system, with a molecular weight of 145kDa [26]. APPL is transported to the axonic terminal were it promotes development of new synapses [30].

In the crab *Chasmaganathus *memory model, repeated presentation of a visual danger stimulus (an opaque screen which moves above the animal) provokes the fading of the initial escape response [31] that is actively replaced by a freezing response. Fifteen or more spaced danger stimuli presentations induce an association between the iterated stimulus and contextual features [32]. A long-term memory is formed (context-signal memory, CSM) which lasts at least for a week and entails protein and mRNA synthesis [33], activation of cAMP dependent protein kinase (PKA) [34,35]), activation of extracellular-signals regulated kinase (ERK) [36] and activation of the NF-κB transcription factor [37,38]. The administration of human β-amyloid peptides induces amnesia in the crab CSM that is dependent of the level of aggregation of the peptide [22].

Here we partially cloned and sequenced a beta-amyloid precursor protein-like gene homologue in the crab *Chasmagnathus granulatus *(cAPPL) and we studied its expression in the central nervous system and other tissues. We also analyzed the time course of gene and protein expression during long-term memory consolidation, observing a different expression profile compared to a passive control group. Our results suggest a role of cAPPL during long-term memory formation.

## Results

### Partial cDNA cloning and sequencing of cappl

**Figure 1:**
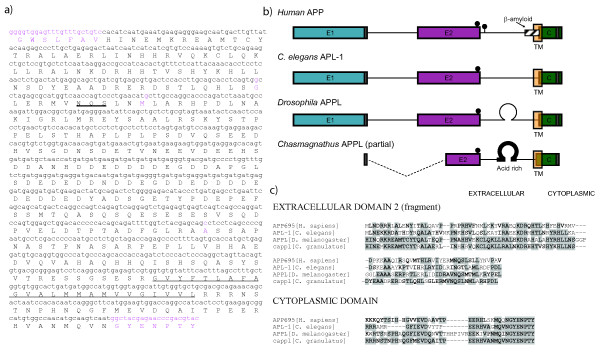
**cAPPL sequence and comparison with other APP-like proteins**. **a) **Partial nucleotide and deduced aminoacid sequence for cAPPL. The predicted transmembrane region is double underlined and a posible glycosilation signal is underlined in bold. Bases corresponding to degenerate primer regions in the sequencing or different in one of the three sequenced clones are highlighted in violet. **b) **General characteristics of APP family members. Large boxes represent the conserved extracellular (E1, E2) and cytoplasmic conserved regions (C). Yellow boxes are indicative of putative transmembrane domains. N-Glycosylation sites (NXS/T with X≠P) are indicated with balloons. Dark gray boxes indicate regions of absolute sequence identity used for degenerate primer design. **c) **Aminoacid sequence comparison. The regions of highest homology between human, C. elegans, Drosophila and Chasmagnathus are presented. Shaded and bold residues are identical, bold aminoacids represent conservative changes.

**Figure 2:**
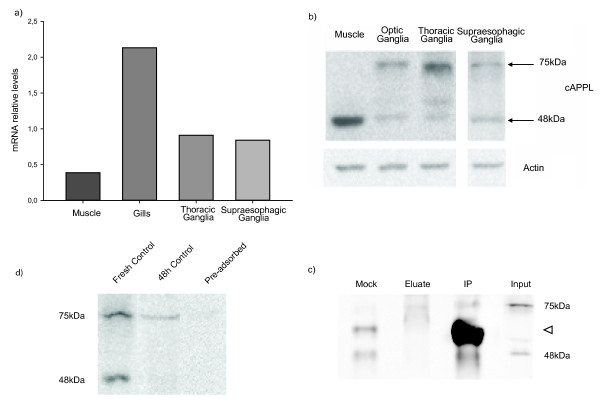
**Presence of cAPPL in central nervous system and other tissues**. **a) **Relative levels of mRNA in different tissues. **b) **Western blot of protein levels in different tissues using an antibody against APP (upper) and actin (lower). **c) **Western blot of the pre-adsorption assay. Fresh Control, antibody non-incubated; 48 h Control, antibody incubated without peptide; Pre-adsorbed, antibody incubated with the peptide. **d) **Western blot of the immunoprecipitation using an antibody against APP. Mock control corresponding to immunoprecipitation with preimmune rabbit serum. The arrowhead indicates the 55kDa band corresponding to the heavy chain of the antibody.

We used a degenerate primer strategy to clone the cDNA of the putative APP homologue in the crab *Chasmagnathus granulathus *and obtained a 1126 bp fragment that codes for 376 aminoacids (Figure 1a), [GenBank accession number: FJ666122] similar to APP or APP-like proteins in both invertebrates (*D. melanogaster*, *C. elegans*) and vertebrates (*H. sapiens*, *P. troglodytes*, *M. musculus*, *R. norvegicus*, *C. familiaris*, *S. scrofa*, *G. gallus*, *X. tropicalis*, *D. rerio*). Given that the estimated size of full length cAPPL is 75kDa (Figure 2b), the partial cDNA obtained codes for approximately 60% of the protein sequence (Figure 1b). The sequenced fragment is consistent with a type I protein with a short cytoplasmic tail, a hydrophobic transmembrane region and a long extracellular domain. The regions not sequenced are a portion of the N-terminal extracellular region and a short stretch of the C-terminal cytoplasmic tail.

We aligned our fragment of cAPPL with *H. sapiens *APP_695_, *D. melanogaster *APPL and *C. elegans *APL-1 using ClustalW 1.82 [39] (Figure 1c). There were both similarities and important differences with other members of the APP-like family. As expected, sequence similarity of cAPPL was highest with *D. melanogaster *APPL (37% identity vs 24% with *C. elegans *APL-1 and 23% with *H. sapiens *APP_695_). There is no observed sequence similarity with the β-amyloid peptide region present of vertebrates (only 11.9% identity with 4 of 5 identical residues in the transmembrane region), a result consistent with its absence in the invertebrate APP genes sequenced to date. As in other invertebrates, there are certain regions with a much higher degree of sequence similarity. Using the same criteria as [29], we assessed sequence identity in the E2 extracellular region (62% with *D. melanogaster *APPL, 37% with *C. elegans *APL-1, 31% with *H. sapiens *APP_695_), the C cytoplasmic region (65% with *D. melanogaster *APPL, 61% with *C. elegans *APL-1, 53% with *H. sapiens *APP_695_) and the sequence between E2 and the membrane spanning region (15% with *D. melanogaster *APPL, 10% with *C. elegans *APL-1, 11% with *H. sapiens *APP_695_). The fragment of cytoplasmic domain sequenced includes a putative G_0_-binding domain (60% sequence identity with equivalent region in *Drosophila*). Additionally, there is a conserved N-glycosylation site present towards the C-terminal end of the E2 domain.

There are also important differences between cAPPL and other members of the APP family. Notably, the entire region between conserved extracellular domains E1 and E2 is missing, as is the N-terminal region of E2. Though this might be due to alternative splicing in the supraesophagic ganglion (central brain), we assessed several other tissues (thoracic ganglion, eye stalks, muscle, and gills) and none of them had higher molecular weight cAPPL cDNAs. This allows us to speculate that the cAPPL gene is indeed missing the corresponding coding regions. The other major difference is an acid rich domain present in cAPPL between E2 and the transmembrane region (in a 69 residue stretch, 67% (46) are acid), also present in *D. melanogaster *APPL.

### Characterization of cAPPL expression

We studied *cappl *gene expression using Real Time PCR. In a first approach we determined the gene expression pattern in different tissues: gills, muscles and two areas of the CNS: supraesophageal and thoracic ganglia. We found that *cappl *is expressed in all tissues analyzed, with higher expression in gills, lower expression in muscles and similar intermediate expression in the nervous tissues (Figure 2a).

We went on to analyze the presence of cAPPL protein product in the central brain and in different tissues of *Chasmagnathus*. We employed a commercial antibody directed against the C-terminal ending of Human-APP protein in Western blot assays. This antibody recognizes a highly conserved region of the protein, parts of which are present in the cloned fragment of the crab *Chasmagnathus*. (K**MQ**Q**NGYENPTY**KFFEQMQN, residues conserved in crab in bold). We found the protein in all tissues studied. In nervous tissues (optic, thoracic and supraesophageal ganglia) we detected two bands of 75kDa and 48kDa, respectively. In contrast, we found a high level of the 48kDa band in muscle but we did not detect the 75kDa band (Figure 2b). Pre-adsorption of the antibody with a peptide containing the conserved sequence MQQNGYENPTY showed an important reduction of both, 75kDa and 48kDa signals (Figure 2c). We also performed immunoprecipitation assay (IP) with this antibody from supraesophageal ganglia extracts. We found the presence of both bands in the IP but the 48kDa band was also found in the mock control (Figure 2d), suggesting that the 75kDa band corresponds to specific recognition of cAPPL but the 48kDa band, does not. Thus, in the experiments performed to study protein levels only the 75kDa band was analyzed.

**Figure 3:**
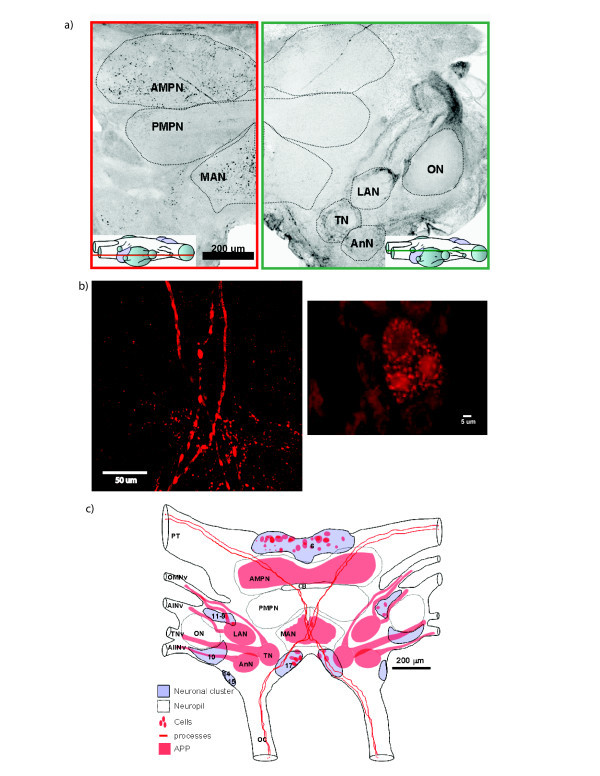
**Immunohistochemistry of central brain cAPPL**. **a) **Stain found in neuropiles and tracts at different depth **b) **Detail of the MAN medial region where stained tracts form a chiasm (left) and detail of cells found in the chiasm region of a slice (right), showing a granular distribution in cytoplasm and a nuclear staining, present only in some of the stained cells. **c) **Map of regions where staining was found. **a **and left **b **are wole mounts. Right **b **is a section.

We then studied cAPPL protein localization in the central brain of *Chasmagnathus *by immunofluorescence of naïve animals in whole mount and sections. Representative images obtained by confocal microscopy are presented in Figure 3. We found positive labeling in different areas of the brain. The labeling shown was bilateral and no stain was found in controls without primary antibody (data not shown). The signal is appreciable in both neuropiles and neuronal clusters. In neuropiles the staining appears at processes and structures resembling synapses, and in the neuronal clusters the staining appears in the cytoplasm and nucleus. The protein distribution includes all three main divisions of the brain protocerebrum, deutocerebrum and tritocerebum, but showing a particular strong localization in four symmetrical processes, three neuropiles with its tracts and two neuropiles with no clear afference. The processes run in the anterior-posterior axis (from protocerebral tracts to the oesophageal connectives). The strongly stained neuropiles are the lateral antenna I neuropil (LAN) in the deutocerebrum, the tegumentary neuropil (TN) and the antenna II neuropil (AnN) in the tritocerebrum. The innervating connectives of all three neuropiles also show strong staining. These connectives are: the oculomotor nerve (OMNv) and antenna I nerve (AINv) that inervate the LAN, the OMNv and the tegumentary nerve (TNv) that innervate the TN and the antenna II nerve (AIINv) that innervate the AnN (Figure 3). The other two stained neuropiles found are the anterior medial protocerebral neuropil (AMPN) in the protocerebrum and the median antenna I neuropil (MAN) in the deutocerebrum. The main somatic staining appears in clusters 6, 11-9 and 17.

### Expression of cappl gene during memory consolidation

**Figure 4:**
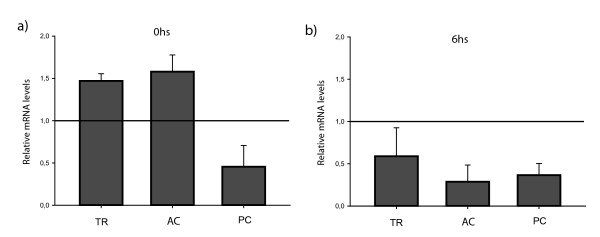
**Time course of cappl gene expression during memory consolidation**: **a) **Mean ± ES of mRNA levels at 0 h after training estimated from two independent experiment. **b**) Mean ± ES of mRNA levels at 6 h after training estimated from two independent experiment. TR, AC and PC groups relative to actin and NV group.

Most studies on the physiological role of APP family are centered on the protein. On the contrary, little is known about the expression and regulation of the APP gene family, particularly in neuronal plasticity and memory processes. To our knowledge, no studies have been performed on the role in memory formation of the APP family in invertebrates. With this in mind, the aim of this section was to determine if there were changes in *cappl *gene expression after LTM formation in the crab CSM paradigm. Four groups were formed. The trained group (TR) had 15 presentations of the visual danger stimulus with an inter-trial interval (ITI) of 171 seconds, the active control group (AC) was subjected to 1012 stimulus presentations with 0 sec ITI and the passive control group (PC) was exposed to the actometer during the same period of time as other groups but no stimulus was presented. A naïve group (NV) was formed with untreated animals to determine basal level of expression of untreated group. Animals were anesthetized, the brains were removed at different time points after treatment and total RNA was extracted. We analyzed relative levels of *cappl *mRNA by Real Time PCR. Figure 4a shows the results obtained immediately after training and Figure 4b shows the results obtained at 6 hs after training. Two way (treatment and time) ANOVA yielded significant differences F(1,8) = 12.17; LSD contrasts, TR-0 vs TR-6, p < 0.05; CA-0 vs CA-6, p < 0.05). We observed an augmentation of *cappl *gene expression in TR and AC groups of about a 50% above NV group immediately after training. On the contrary, a reduction of about 50% was observed in PC. At 6 h, all experimental groups presented a reduction of *cappl *gene expression with respect to NV group.

### cAPPL protein levels during memory consolidation

**Figure 5:**
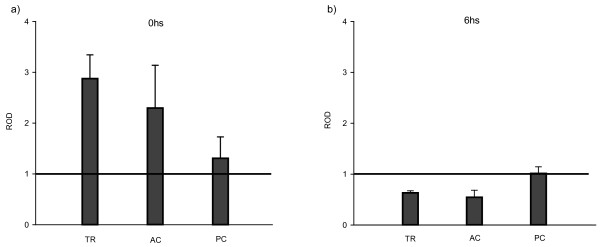
**Time course of cAPPL protein levels during memory consolidation**: **a) **Mean ± ES of protein levels at 0 h after training estimated from two independent experiment. **b**) Mean ± ES of protein levels at 6 h after training estimated from three independent experiment. TR, AC and PC groups relative to actin and NV group.

In this section, we analyzed cAPPL protein levels at different time points after learning. We employed the same groups of animals described above and time points were also coincident in order to compare both results. After the training session, supraesophageal ganglia were obtain and were subjected to a total protein extraction protocol. We performed Western blot assay using anti- C-terminal APP and anti-actin antibodies and the results are expressed as cAPPL levels relative to actin and NV group. Figure 5a shows the results immediately after training. We found an increase in the protein levels in the TR and AC group. Results of protein levels from animals sacrificed 6 h after training are shown in Figure 5b. In this case, TR and AC groups showed a reduced level. PC group levels were similar to NV levels in both time points analyzed (Two factor ANOVA, F(1,3) = 19.43, p < 0.01, interaction F(1,3) = 5,05, p < 0.01; LSD contrasts, TR-0 vs NV-0, p < 0.01; TR-0 vs PC-0, p < 0.05; AC-0 vs NV-0, p < 0.05; AC-0 vs PC-0, p < 0.05; TR-0 vs TR-6, p < 0.01; CA-0 vs CA-6, p < 0.01).

## Discussion and Conclusions

In the present report we partially cloned and sequenced a member of the APP family of proteins in crustacea. Previously, APP members were identified in several vertebrate species, including human, monkey, rat, mouse, frog, fish and four species in invertebrates: the insects *Drosophila melanogaster *(DmAPPL) and *Manduca sexta *(msAPPL), the squid *Loligo pealei *(sAPP) and the worm *Caenorhabditis elegans *(CeAPL) [16,27,28]. The data provided by these sequences, together with our sequence in crab, indicate that APP is a conserved family of transmembrane glycoproteins. Moreover, transgenic studies in Drosophila have shown that human APP695 can rescue at least some of the deficits caused by the deletion of APPL [40], demonstrating that these proteins are functionally as well as structurally conserved.

### Characterization of cAPPL

We first characterized cAPPL gene and protein expression in the central brain and in different tissues of the crab. We found expression of cAPPL mRNA and protein in all tissues analyzed, including the central nervous system. This distribution coincides with that described for different species where genes of this family were studied, excepting *Drosophila*, whose expression is fundamentally in the central nervous system [26]. We only found one fragment by PCR amplification in all tissues studied, suggesting at first that only one transcript is present in this species. However, the fact that we observed only one amplified fragment does not rule out the possibility of an alternative splicing process, if the primers used recognized only one of the variants. The antibody revealed one specific protein in Western blot assay of 75kDa (Figure 2). This protein has a molecular weight similar to that described in mammals and it is smaller than the homologue found in *Drosophila*. We found low mRNA levels in muscle and the protein could not be detect by Western blot, indicating very low expression in this tissue.

Employing the same antibody used in Western blot, we performed immunoflorescence assays and by confocal microscopy we determined cAPPL's presence in different areas of *Chasmagnathus granulatus *central brain. The signal is appreciable in neurites, cytoplasm and nuclei. The presence of the intracellular domain in the nucleus after gamma cleavage of the APP protein was previously reported [41]. The presence of cAPPL in the protocerebrum (AMPN), suggests a role for this protein in neuronal plasticity processes. This neuropil is probably a main associative area where sensory information (mostly visual information) coming from distinct organs is integrated. Recent results from our laboratory indicate this area is involved in neuronal plasticity in CSM (Freudenthal, personal communication). Particularly striking is the presence of four neuritic projections coming from protocerebral tract, through the medial protocerebrum, making a chiasm at the tritocerebrum region, continuing through the oesophageal connectives to thoracic ganglia region (Figure 3). These connections could be involved in information transmission and processing from primary optic areas to the thoracic ganglia region, responsible for motor control responses. As the learning and memory task used is fundamentally visual, these findings suggest a role of cAPPL in the neural circuits that process such information.

### Expression profile of cAPPL during memory consolidation

We analyzed the cAPPL time course of expression at the level of mRNA and protein. We found an increase in mRNA levels immediately after training for the TR group and an inhibition for the PC group. These findings would suggest an involvement of cAPPL in memory formation. However, a similar increment was found in the AC group. The increment of the AC group suggests that this gene is probably regulated by neuronal activity, sensory stimulation, stress and/or motor response. We assume such interpretation taking into account that active controls received stimulus presentation and performed escape responses but were unable to induce LTM formation. The increase of expression found for the TR group could be explained in the same terms, indicating that gene induction is involved in general processes in CSM consolidation. On the contrary, the exposure to the context without stimulation (PC group) reduces *cappl *mRNA levels, whereas protein levels do not change. This result was also found in the 6 hs time point. Indeed, the *cappl *mRNA levels of all experimental groups 6 hours after training where below the NV level. A learning event initiates alteration of gene expression that contributes to synapse remodeling. There have been reports of transcriptional and translational control waves in memory consolidation mechanisms. Multiple gene expression studies using microarrays have proposed a transcriptional inhibition of a wide number of genes between 2 and 6 hours after an inhibitory avoidance learning paradigm. Among the involved genes are molecular adhesion proteins, which could contribute to synapses weakening in the pursuit of synaptic modulation facilitation, so new neuronal circuits can be establish [42]. Keeping in mind that, among other functions, APP could have a role as an adhesion molecule, this transcriptional and translational inhibition 6 hours after training could reflect synaptic weakening.

With regards to the cAPPL protein time course study, there is in general a correlation with gene expression levels.

This is the first report about the APP homologue in a crustacean model. The study of this gene in invertebrate models has a comparative interest and may contribute to the understanding of the physiological role of this protein family in synaptic plasticity and memory and, eventually, could contribute to the understanding of its role in pathology.

## Methods

### Animals

Adult male *Chasmagnathus granulatus *intertidal crabs, 2.6-2.9 cm across the carapace, weight 17 ± 0.2 g (n = 60), were collected from water less than 1 m deep in the estuarine coasts of San Clemente del Tuyú, Argentina, and transported to the laboratory where they were lodged in plastic tanks (30 × 45 × 20 cm) filled to 0.5 cm depth with diluted (12‰, pH 7.4-7.6) marine water (prepared from Cristalsea Marinemix salts, USA), to a density of 20 crabs per tank. The holding room was maintained on a 12 h light-dark cycle (light on 07:00-19:00 h). Temperature of both holding and experimental rooms was maintained at 22-24 °C. Experiments were carried out between the third and the tenth day after the animal's arrival. Experiments were carried out in accordance with the local regulations for the care and use of laboratory animals. All efforts were made to minimize animal suffering and to reduce the number of animals used.

Experiments were performed in accordance with local regulations and the National Institutes of Health (NIH) Guide for the Care and Use of Laboratory Animals (NIH publication 80-23/96). All efforts were made to minimize animal suffering and to reduce the number of animals used.

### Training-testing apparatus

The experimental unit has been described in detail elsewhere [43]. Briefly, it consists of a bowl-shaped plastic container where the crab is lodged and an opaque rectangular screen which moves horizontally above the animal (actometer). Screen displacements evoke a crab's running response and, as a consequence, container vibrations which induce electrical signals through four piezoelectric transducers attached to the external surface of the container. Signals recorded during a trial are translated into numerical units ranging from 0 to 8000. The experimental room has 40 units, separated from each other by partitions. A computer is employed to program trial sequences, trial duration and inter-trial intervals, as well as to monitor experimental events.

A training session consisted of 15 trials with an inter-trial interval (ITI) of 171 sec. Each trial lasted 9 sec and consisted of two cycles of presentation of the screen over the actometer. Each cycle lasted 2.5 sec with a 2 sec interval between cycles (TR group). Crabs' activity was recorded during the entire trial time. For continuous training (AC group), animals were subjected to 1012 stimulus presentations without ITI. The passive control group (PC) was exposed to the actometer during the same period of time as the other groups but no stimulus was presented. Finally, the naïve group (NV) was not exposed to the actometer. Animals were placed in the actometers 10 min prior to the start of the experiment to allow adaptation to the device.

### Cloning of cappl

We designed degenerate primers based on two sequences highly conserved in vertebrate and invertebrate taxa: GVEFVCCP (zinc binding domain) and NGYENPTY (involved in clathrin attachment and internalization) [16]. Primer sequences were 5'-GGNGTNGARTTYGTNTGYTGYCC-3' (APP1) and 5'-RTANGTNGGRTTYTCRTANCCR-3' (APP2) respectively. We obtained total RNA from the supraesophagic ganglion of adult, male *Chasmagnathus granulathus *using the protocol described by [44]. After reverse transcription (Promega), the cDNA was subjected to PCR (*Taq*, Invitrogen): 2 min at 95°C, 1 min at 95°C, 1 min at 54.6°C, 2 min at 72°C (40 cycles), and 5 min at 72°C. A resulting band of about 1 kbp was excised from an agarose gel, column purified and subjected to 40 more cycles of PCR using the same parameters. The reamplified band of about 1 kbp was excised from an agarose gel, column purified and cloned into a pGEM-T Easy vector (Promega). Three clones were sequenced from either side of the multiple cloning site (Macrogen) and yielded an insert of 1126 bp (including primers) almost identical between clones. Where necessary, we considered the correct base to be that shared by two of the three clones sequenced.

### Sequence analysis

We used NCBI's Basic Local Alignment Tool (BLAST) to search for similarity between the translated sequence derived from the cDNA and proteins present in databases (blastx). For further comparison to members of the APP family we used ClustalW 1.82 with default settings.

### RNA extraction and quantification by Real Time PCR

Animals were anaesthetized by immersion in ice-cold water for two min. The central brain was then dissected. Twenty ganglions per sample were pooled in liquid nitrogen. RNA extraction was carried out by a standard method (TRIZOL^® ^Reagent, INVITROGEN). We used equal amounts of RNA between groups (assessed by gel electrophoresis) for reverse transcription reactions (*Promega*). Equal amounts of the resulting cDNA were used as templates in Real Time PCR (*DNA Engine Opticon System*). To amplify a fragment of *cappl *we used the following primers, forward: cacccagatctaaatgccaag; backward: ggagcatgtgtggacagttc, which amplify a 101 bp fragment. To amplify a fragment of actin we used the following primers, forward: tcctgggtatggaatccgttgg; backward: gtctggccccaccaccatgtac, which amplify a 122bp fragment. Reaction conditions for APP were as follows: 1 μl template, 2 mM Mg^2+^, 50 nM dNTPs, 2.5 μM of APP primers, 5% DMSO with SYBR Green (*Roche*) and Taq buffer and polymerase (*Invitrogen*) in 20 μl reaction volume. Reaction conditions for actin were as follows: 0.5 μl template, 2 mM Mg^2+^, 0.2 mM dNTPs, 1 μM of actin primers, 5% DMSO with SYBR Green (*Roche*) and Taq buffer and polymerase (*Invitrogen*) in 20 μl reaction volume. Cycling conditions were: 3 min at 94°C, 40× (45 s at 93°C, 30 s at 56°C, 1 min at 72°C, with plate read) and 10 min at 72°C. After each experiment, melting curves were assessed to confirm the absence of significant amounts of primer dimers or nonspecific amplifications.

### mRNA Quantification

*cappl *mRNA levels relative to the naïve group and actin values of all experimental groups were quantified using the following equation [45]:

$$mRNA\;relative\;level=\frac{\text{cAPPL}\;{\text{efficiency}}^{\text{(Ct Naïve-Ct}\;\text{group)}}}{\text{Actin}\;{\text{efficiency}}^{\text{(Ct Naïve-Ct}\;\text{group)}}}$$

Actin efficiency (Ct Naïve-Ct group) where Ct is threshold cycle value obtained by Real Time PCR and the efficiency was calculated from a calibration curve for both *cappl *gene and actin gene. The efficiency of *cappl *amplification was 1.88 (R^2 ^= 0.99) and the efficiency of actin amplification was 2 (R^2 ^= 0.99).

### Protein extraction and Western blot assay

Animals were anaesthetized by immersion on ice-cold water for two min. The central brain was then dissected. Twenty ganglions per sample were pooled in 1 ml buffered crab saline solution (pH 7.6). Total protein extracts were obtained as follows. Dissected supraesophageal ganglia were homogenized (ULTRA TURRAX^® ^T25 basic, IKA Labortechnik) in 300 μl buffer A (Hepes 10 mM pH 7,9, MgCl 1,5 mM, KCl 10 mM, DTT 1 mM) with proteases inhibitors (PMSF 0,5 mM, Pepstatin A 1 ug/ml, Leupeptin 10 μg/ml, Aprotinin 10 μg/ml) and centrifuged at 3000 G for 5 min at 4°C. Supernatant was stored at -20 until use. Protein content of extracts was measured via Bradford assay.

For the Western blot assay, loading buffer was added and samples were incubated at 100°C for 5 min. 20 μg of protein was run in 10% SDS-PAGE with 15% polyacrylamide in the resolving gel. Proteins were then electro-transferred to a PVDF membrane for immunoblotting against cappl (Sigma A 8717, Lot# 086K4858) and actin. The antibody against cappl recognizes the highly conserved C-terminal portion of the protein KMQQNGYENPTYKFFEQMQN. This antibody was incubated ON in TTBS with 5% non-fat milk. Detection was performed with Luminol chemiluminiscence kit (SCB) as described by manufacturer and the signals were digitalized (FUJIFILM-Intelligent Dark Box II apparatus with image reader LAS-1000 software). The relative optical density (ROD) was meassured using NIH-ImageJ 1.29× software. The protein levels was relativized to actin ROD and then relativized to the mean of the NV group.

The pre-adsorption of the antibody was performed adding 250 ug/ml of the conserved peptide to the TTBS dilution of the antibody without non-fat milk and incubated 48 h at 4°C previously to be used in western blot.

### Immunohistochemistry

Dissected supraesophageal ganglia of naïve animals were fixated 2 h with para-formaldehyde (PFA) 4% in 0.1 M saline phosphate buffer (PBS), pH 7.2. After washing the tissue 10 minutes in PBS, ganglia were mounted in low melting agarose and sliced with a vibratome in 200 μm transverse sections (sections). Another group of ganglia (Whole Mounts) were homogenously perforated with a microelectrode of ≈2 μm of tip diameter. Then both groups were blocked in PBS with 1% Triton-X100 and 2% bovine serum albumin (BSA), 4 h while shaking. After a 5 min wash in PBS, the anti-C-terminal APP antibody (Sigma) was incubated overnight at 4°C in PBS with 0.3% Tween 20 at a dilution of 1:500. After a 5 min wash with PBS, ganglia were incubated at 4°C for 5 hs in a solution with secondary antibody IgG-rodamine against the Fc region of rabbit IgG in a 1:500 dilution in PBS. Tissues were washed once in PBS for 5 min, twice in PBS with 1% Triton-X100, once in PBS with 0,3% Tween 20 overnight and a last time 10 min in PBS. Ganglia were placed in PBS with 50% glycerol for 5 min and mounted in a PBS with 80% glycerol solution for observation by confocal microscopy (Olympus FV300). Images were acquired by FluoView software, and analyzed with NIH-ImageJ 1.29×. The anatomical terminology used for brain structures was taken from [46].

### Immunoprecipitation

50 μl of Protein A/G PLUS-Agarose immunoprecipitation reagent (Santa Cruz Biotechnology, INC. sc-2003) were washed with 200 μl of PBS and spun down at 5000 g, discarding the supernatant. This procedure was repeated two times. The pellet was divided into two tubes: one incubated with 2 μl of preimmune rabbit serum (mock) and the second one with 3 μl of the same antibody against APP used for western blot (IP). Both were incubated overnight at 4°C. Both tubes were washed three times as described above. The mock tube was incubated 1 hr at room temperature with 5% glycerol, 0.3% tween, PBS, 500 μg of the protein extracted and protease inhibitors. After a spin down, the supernatant was transferred to the IP tube. The IP tube was incubated overnight at 4°C. The supernatant obtained after a spin down was transferred to another tube (eluate). IP tube and mock tube were wash two times with PBS, 5% glycerol and 0,3% Tween and twice with Buffer USA (50 mM Tris, 5 mM EDTA, 250 mM NaCl, 50 mM NaF, 0,1% Triton, 0,1% Na_3_VO_4_, 5% Glycerol); the last wash was with PBS alone. The samples were analyzed by western blot as described above.

## Abbreviations

CAPPL: *Chasmagnathus granulatus *amyloid precursor protein like gene; CAPPL: *Chasmagnathus granulatus *amyloid precursor protein like protein; βA: β- amyloid; NV: Naïve untreated crabs; TR: Spaced Trained crabs; AC: Active Control; PC: Passive Control; IP: Immunoprecipitated.

## Authors' contributions

MSF Performed behavioral task, ganglia extraction, mRNA and protein extraction, Western Blot, Real Time PCR, immunoprecipitation assay and immunohistochemistry assays. Contributed with the data analysis and writing of the manuscript. PA Cloned the partial fragment of cappl and participated with optimization of Real Time PCR technique. Contributed with the writing and language correction of the manuscript. NF Participated with all ganglia extractions and with Real Time PCR technique. RF Participated with the performance, analysis and description of the immunohistochemistry assay and the immunoprecipitation assay. Contributed with the writing of the manuscript. AR Directed the project, contributed with the data analysis and writing of the manuscript. All authors read and approved the final manuscript.
